# Awake state-specific suppression of primary somatosensory evoked response correlated with duration of temporal lobe epilepsy

**DOI:** 10.1038/s41598-020-73051-x

**Published:** 2020-09-28

**Authors:** Makoto Ishida, Kazutaka Jin, Yosuke Kakisaka, Akitake Kanno, Ryuta Kawashima, Nobukazu Nakasato

**Affiliations:** 1 Department of Epileptology, Tohoku University Graduate School of Medicine, 2-1 Seiryo-machi, Aoba-ku, Sendai, Miyagi 980-8575 Japan; 2 Collaborative Laboratory of Electromagnetic Neurophysiology, Tohoku University Graduate School of Medicine, Sendai, Miyagi Japan; 3 Institute of Development, Aging and Cancer, Tohoku University, Sendai, Miyagi Japan

**Keywords:** Neuroscience, Neurology

## Abstract

Epilepsy is a network disease. The primary somatosensory cortex (S1) is usually considered to be intact, but could be subclinically disturbed based on abnormal functional connectivity in patients with temporal lobe epilepsy (TLE). We aimed to investigate if the S1 of TLE is abnormally modulated. Somatosensory evoked magnetic fields (SEFs) evoked by median nerve stimulation were recorded in each hemisphere of 15 TLE patients and 28 normal subjects. All responses were separately averaged in the awake state and light sleep using background magnetoencephalography. Latency and strength of the equivalent current dipole (ECD) was compared between the groups for the first (M1) and second peaks. Latencies showed no significant differences between the groups in either wakefulness or light sleep. ECD strengths were significantly lower in TLE patients than in controls only during wakefulness. The reduction of M1 ECD strength in the awake state is significantly correlated with duration of epilepsy. SEFs of TLE patients showed pure ECD strength reduction without latency delay. The phenomenon occurred exclusively during wakefulness, suggesting that a wakefulness-specific modulator of S1 is abnormal in TLE. Repetitive seizures may gradually insult the modulator of S1 distant from the epileptogenic network.

## Introduction

Epilepsy is a disease characterized by disturbance of the excitatory-inhibitory synaptic balance in the epileptogenic network (EN)^1,2^. Abnormal neuronal imbalance occurs in the temporal and connected regions in temporal lobe epilepsy (TLE)^3^. The primary auditory area and auditory association areas may be included in the EN of patients with TLE^4^. Consequently, the auditory-evoked response is useful for evaluating cortical activities in functional areas. Recent studies using auditory-evoked magnetic fields reported physiological changes in auditory function in patients with mesial TLE^5–7^. The functional connectivity of the ascending reticular activating system (ARAS) may also be disturbed inside the EN in patients with TLE. Recent functional magnetic resonance (MR) imaging studies revealed that activity of the ARAS was negatively correlated with increased frequency of focal impaired awareness seizures^8^, and/or neurocognitive problems^8,9^. The ARAS pathway is crucial in maintaining arousal and consciousness^10–12^. The ARAS widely projects to the cerebral cortex including the primary somatosensory cortex (S1) from the reticular formation in the brainstem^13^, and modulates cortical function through the balance between excitatory and inhibitory synaptic transmissions mainly in the awake state^10,14,15^.

The S1 is usually considered to be outside the EN in patients with TLE. The primary somatosensory function is believed to be normal in TLE, but could be subclinically disturbed based on the abnormal functional connectivity described above. The N20m response (M1 in the present study) to median nerve stimulation, the first cortical somatosensory evoked field (SEF) peak and its counterpart somatosensory evoked potential (SEP) N20, is an established marker of primary somatosensory function^16,17^. The N20m/N20 is a tangential current to the scalp generated at the posterior bank of the central sulcus, so the equivalent current dipole (ECD) of N20m is suitable for quantitative evaluation because magnetoencephalography (MEG) is exclusively sensitive to cortical tangential activity^16–18^. Amplitude enlargement of N20m during sleep may occur in healthy subjects^19,20^, but remains unevaluated in patients with neurological disease.

The present study evaluated median nerve SEFs in the awake state and light sleep of patients with TLE to clarify whether the excitation level is abnormal in the S1 distant from the EN.

## Results

**Figure 1:**
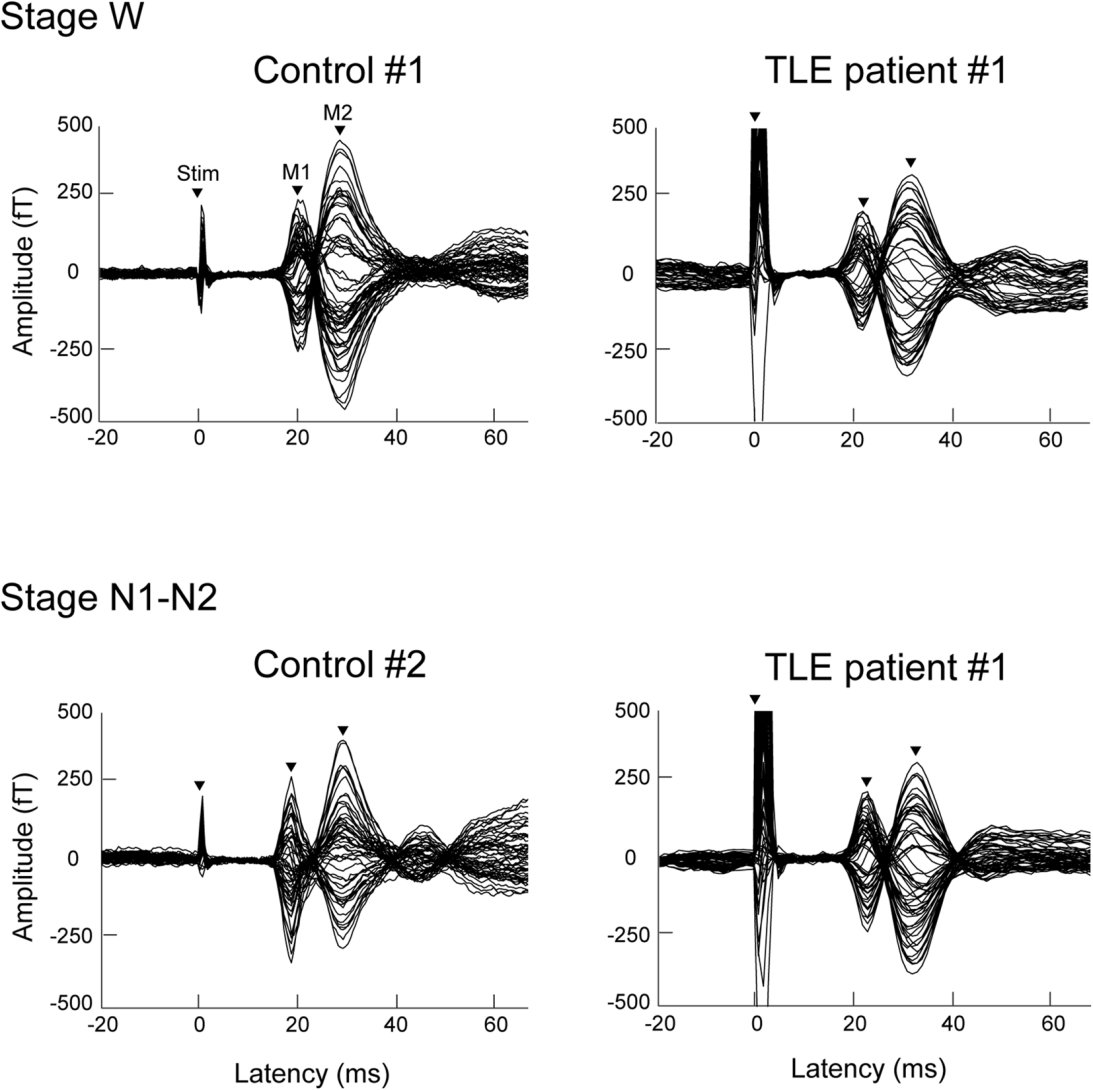
Typical examples of somatosensory evoked field (SEF) waveforms in temporal lobe epilepsy (TLE) patients and normal subjects. Stacked waveforms of selected magnetoencephalography channels showing the SEFs over the contralateral hemisphere induced by median nerve stimulation at the wrist. Every single evoked response was separately averaged for the awake state (stage W) and light sleep (stage N1–N2). Note smaller amplitudes of first (M1) and second (M2) peaks of SEF in TLE patients than in normal subjects in the awake state but not in light sleep.

Figure 1 shows typical examples of SEF waveforms during awake state and light sleep in a patient with TLE and two normal subjects. The amplitudes of M1 and M2 were smaller in TLE patients than in normal subjects in the awake state but not in light sleep.

### Comparison of latency between TLE patients and normal subjects

**Figure 2:**
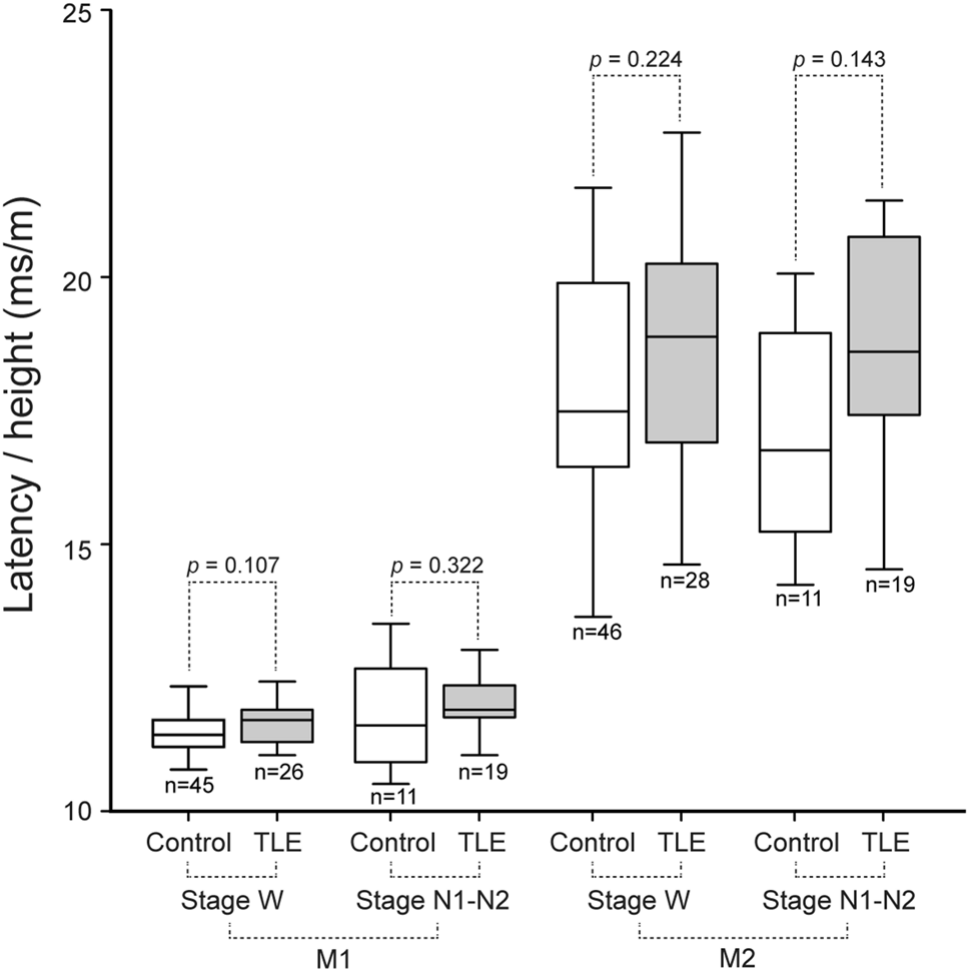
Comparison of somatosensory evoked field (SEF) latency between temporal lobe epilepsy (TLE) patients and normal subjects. Latency normalized by height at the first (M1) and second (M2) peaks of SEFs induced by median nerve stimulation was compared between TLE patients and normal subjects, during both the awake state (stage W) and light sleep (stage N1–N2). Box-and-whisker plots show minimum, 1st quartile, median, 3rd quartile, and maximum values from the bottom to the top. No significant differences were found in latency at the M1 and M2 of SEFs between TLE patients and normal subjects, during both the awake state and light sleep.

No significant differences were found in latency of the M1 and M2 peaks of SEFs between TLE patients and normal subjects, during both the awake state and light sleep (Fig. 2).

### Comparison of ECD strength between TLE patients and normal subjects

**Figure 3:**
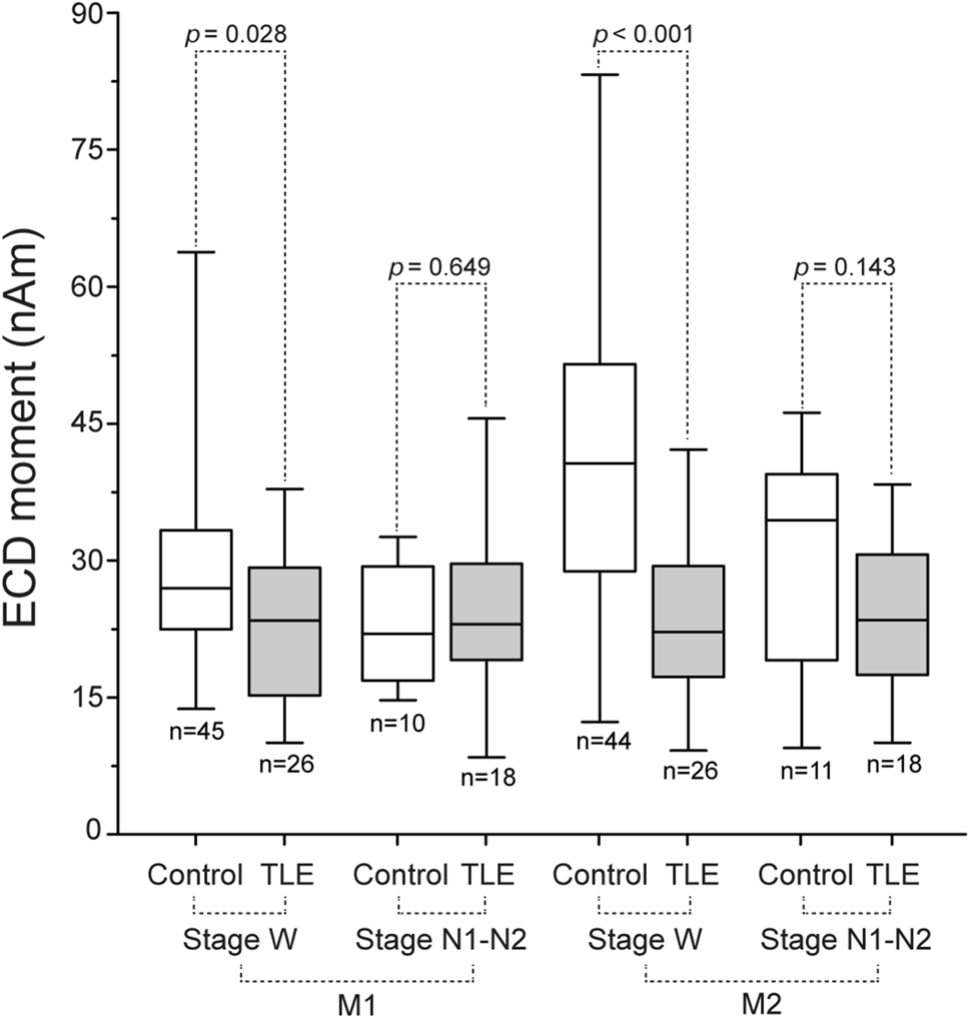
Comparison of somatosensory evoked field (SEF) strength between temporal lobe epilepsy (TLE) patients and normal subjects. Equivalent current dipole (ECD) strength at the first (M1) and second (M2) peaks of SEFs induced by median nerve stimulation was compared between TLE patients and normal subjects, during both the awake state (stage W) and light sleep (stage N1–N2). Box-and-whisker plots show the minimum, 1st quartile, median, 3rd quartile, and maximum values from the bottom to the top. ECD strengths at the M1 and M2 were significantly lower in TLE patients than normal subjects during the awake state (*P* = 0.03 and *P* < 0.001, respectively, two-sided Mann–Whitney U test), but not during light sleep.

ECD strengths at the M1 and M2 were significantly lower in TLE patients than in normal subjects during the awake state (*P* = 0.028 and *P* < 0.001, respectively, two-sided Mann–Whitney U test), but not during light sleep (Fig. 3).

### Correlation between M1 ECD strength in the awake state and variables

**Figure 4:**
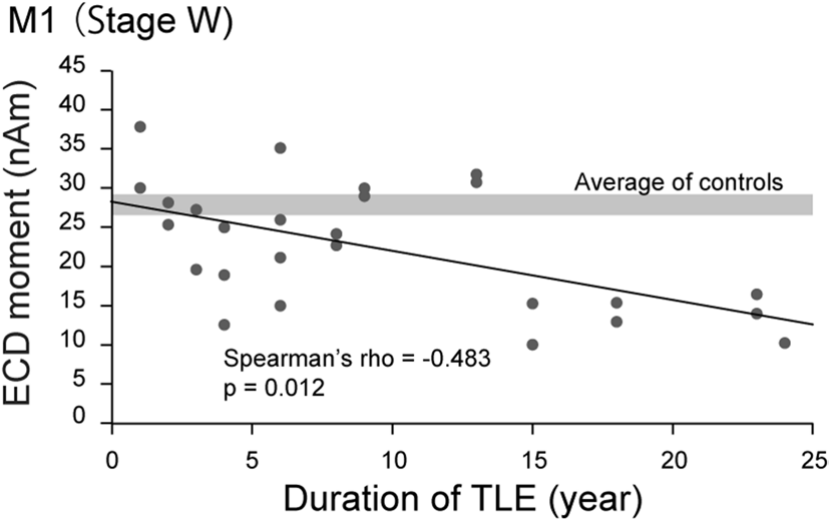
Correlation between strength in the awake state and duration of epilepsy in temporal lobe epilepsy (TLE) patients. Reduction of equivalent current dipole (ECD) strength at the first peak (M1) of somatosensory evoked fields in the awake state was correlated with duration of epilepsy in TLE patients. Note the M1 strength of the approximate line at seizure onset (0 year) was almost equal to the average of normal subjects.

The correlations between M1 ECD strength in the awake state and clinical variables are summarized in Table 1. No significant correlations were found between M1 ECD strength and age, sex, side of TLE, presence of lesion, presence of hippocampal sclerosis (HS), and relationship to epileptic focus. Lower M1 ECD strength in the awake state was significantly correlated with duration of epilepsy in TLE patients (Spearman’s *ρ* = − 0.483., *P* = 0.012) (Fig. 4). M1 strength of the approximate line at seizure onset (0 year) was almost equal to the average for normal subjects.

**Table 1:** Correlation between M1 ECD strength in the awake state and clinical variables in TLE.

Clinical variables	*P*-value
Age (n = 26)	0.747 (0.066)*
**Sex**	
Male (n = 14)	0.898
Female (n = 12)	
**Side of TLE**	
Left (n = 15)	0.350
Right (n = 11)	
**Presence of lesion**	
Lesion (n = 20)	0.287
Non-lesion (n = 6)	
**Presence of HS**	
HS (n = 13)	0.608
Non-HS (n = 13)	
**Relationship to epileptic focus**	
Affected hemisphere (n = 13)	0.798
Unaffected hemisphere (n = 13)	
Duration of TLE (n = 26)	0.012 (− 0.483)*

*M1* first peak of the somatosensory evoked field, *ECD* equivalent current dipole, *TLE* temporal lobe epilepsy, *HS* hippocampal sclerosis.

*Spearman’s ρ correlation coefficient test, *P*-value (rho).

## Discussion

The present study revealed that the ECD strength of SEFs was significantly lower in patients with TLE than in normal subjects exclusively during the awake state without prolongation of latency. Moreover, the lower M1 ECD strength in the awake state was correlated with duration of epilepsy. These results imply that cortical function even outside the EN is abnormal in TLE patients as a function of epilepsy duration.

First, no significant differences were found in SEF latencies between the TLE and control groups. SEP and SEF latencies are prolonged if nerve conduction is affected at any level of the somatosensory pathway from the peripheral nerves, spinal cord, and brainstem to the cortex^21–23^. Therefore, the absence of abnormality in the nerve conduction pathway to the S1 was confirmed. Nevertheless, lower SEF ECD strength was detected in only the TLE patients exclusively during the awake state. Some awake state-specific modulators must be abnormal at the S1 in TLE to cause such reduction of M1 and M2 ECD strengths during the awake state but not during light sleep.

The ARAS may be involved in at least some of the present findings of SEF abnormalities during awake state, because the ARAS is more active in the awake state than during sleep^14,24^. Therefore, disturbance of the ARAS may have resulted in the suppression of primary SEF magnitude in our TLE patients during the awake state. Previous investigations of SEP^25^ and SEF^19^ revealed decreased high frequency oscillations in the S1 during sleep, suggesting decreased function of the inhibitory neurons.

Pure amplitude reduction without latency delay of N20/N20m is an extremely rare phenomenon, reported only in a few cases of direct cortical damage such as in Creutzfeldt-Jakob disease^26^. Amplitude reduction of N20/N20m is usually associated with latency delay as occurs in multiple sclerosis^23^, brain lesions, and severe head injury^21,22^. Greatly increased SEP and SEF, often named “giant SEP and SEF,” are frequently reported in epilepsy with cortical myoclonus^27–30^ and traumas^21^. However, amplitude enlargement occurs only in the later components such as M2, but not in N20m (M1) or N20. The present results are quite unique because M1 (N20m) showed “pure amplitude reduction without latency delay,” representing pure dysfunction at the level of the S1.

One important finding of the present study is the new fact that M1 ECD magnitude in the awake state is inversely correlated with duration of epilepsy. Deterioration of an awake state-specific modulator of the S1, such as ARAS, may start at the clinical onset of TLE, because the M1 magnitude of the approximate line at seizure onset (0 year) is almost equal to the average for normal subjects. A previous functional MR imaging study revealed that ARAS connectivity was significantly lower in patients with TLE than in controls, particularly in those with neocortical regions^8^. The diminished ARAS connectivity was related to increased frequency of focal impaired awareness seizures, which are associated with impairments in verbal intelligence quotient, attention, executive function, language, and visuospatial memory on neuropsychological evaluation^8^. Therefore, repetitive seizures in TLE, but not preceding pathology, are likely to gradually insult an awake state-specific modulator of the S1 distant from the EN.

Antiepileptic drugs (AEDs) may modify SEF, due to the effect on the synaptic neurotransmission and the modification of cortical excitability. Recent studies reported no significant differences^31–33^ or prolonged latency^34^ in SEP during treatment with AEDs. Therefore, AEDs are not considered to cause significant change in the amplitude of SEP.

Previously, separation of the awake and sleep recordings for SEP or SEF analysis was conducted in only normal subjects^20,35–37^. Our present method involving strict separation of the awake and sleep stages by visual assessment of background MEG could also be used to evaluate cortical excitation in the somatosensory areas distant from the EN in other types of epilepsy. Moreover, this novel method will be useful for further studies to evaluate cortical excitation in various types of neurological diseases.

The present study has the limitation of small sample size of SEF data during light sleep. MEG recordings with the head inside the helmet during repetitive electrical stimulation may have prevented some subjects falling asleep. Further studies including more data during light sleep are needed to confirm our results.

## Materials and methods

### Participants

The present study included 15 patients with TLE (8 males; age 20–29 years, mean 25.0 years) who underwent comprehensive evaluation for epilepsy at Tohoku University Hospital Epilepsy Monitoring Unit, Sendai, Japan from January 2015 to December 2017, as well as 28 healthy volunteers (19 males; age 20–27 years, mean 22.4 years). The patients were electro-clinically diagnosed with TLE based on the clinical history and the results of long-term video EEG monitoring. Patients with multi-lobar epilepsy, such as frontal or parietal lobe epilepsy combined with TLE, were excluded. None of the TLE patients had structural abnormalities in the primary somatosensory cortex, as verified by MR imaging. The clinical characteristics of the patients are summarized in Table 2.

**Table 2:** Clinical characteristics of 15 patients with TLE.

Case no.	Sex/age (years)	Epilepsy duration	Side of TLE	Etiology	Seizure type		AED
					FIAS	FBTCS	
1	M/23	1	Left	HS	+	+	CZP
2	M/23	2	Left	Ganglioglioma	+	+	CBZ, LEV
3	F/28	3	Right	Amygdala enlargement	+	−	CBZ, LEV, PHT, CZP
4	M/26	4	Left	Tumor	+	+	CBZ, LEV
5	M/20	4	Right	Ganglioglioma	+	+	CBZ, LEV
6	M/22	6	Left	HS	+	+	LEV, ZNS
7	F/25	6	Left	HS	+	+	LEV, LTG, CLB
8	F/29	6	Right	Viral encephalitis	+	+	CBZ
9	F/20	7	Left	HS	+	−	CLB, ZNS
10	M/29	9	Left	Unknown	+	+	None
11	F/23	13	Left	HS	+	−	CBZ
12	F/29	15	Right	Unknown	+	+	CBZ, LEV, LTG
13	F/23	18	Left	HS	+	−	CBZ, LEV, CLB, TPM
14	M/28	23	Right	Unknown	+	+	VPA, LEV
15	M/27	24	Left	HS	+	−	CBZ, ZNS

*AED* anti-epileptic drug, *CBZ* carbamazepine, *CLB* clobazam, *CZP* clonazepam, *F* female, *FBTCS* focal to bilateral tonic–clonic seizure, *FIAS* focal impaired awareness, *HS* hippocampal sclerosis, *LEV* levetiracetam, *LTG* lamotrigine, *M* male, *PHT* phenytoin, *TPM* topiramate, *ZNS* zonisamide, *VPA* valproic acid.

The study was approved by the ethical committee of Tohoku University Graduate School of Medicine (2010-189, 2012-1-459, 2017-2-102-1) and written informed consent was obtained from all patients or legally authorized representatives and subjects in accordance with the requirements of the ethical committee. All parts of the present study were performed in accordance with the guidelines of the Declaration of Helsinki (1991).

### MEG recordings with median nerve stimulation

MEG was performed with a 200-channel whole-head MEG system with axial gradiometers (RICOH Ltd., Tokyo, Japan) in a magnetically shielded room. The detailed conditions of the MEG system used in this study have already been described^38,39^. Briefly, the sensors were first-order axial gradiometers with a baseline of 50 mm and 15.5-mm diameter coils. The sensors were arranged in a uniform array over a helmet-shaped surface at the bottom of a Dewar vessel. The centers of two adjacent coils were separated by a mean distance of 25 mm.

All patients and subjects lay in the supine position, with the head location determined by the positions of five fiduciary markers consisting of induction coils placed at known locations on the scalp. The head shape and coil positions were established using a three-dimensional digitizer (FastSCAN Cobra, Polhemus, Inc., Colchester, VT) based on three-dimensional MR images obtained for all patients and subjects using a 3T MR system (Achieva, Philips Healthcare, Best, the Netherlands; or Magnetom Trio, Siemens AG, Erlangen, Germany)^38,39^.

The left and right median nerves at the wrist were stimulated independently by constant-current stimuli of biphasic square-wave impulses with 0.3 ms duration at 0.7 Hz^21,40^. Stimulus intensity was set at 1.5 times the motor threshold to evoke a twitch of the thumb^21^. The conditions for data acquisition with the MEG systems were sampling at 1000 or 2000 Hz and low-pass filtering at 100, 200, or 500 Hz in each patient or subject.

### Analysis of SEFs in the awake state and light sleep

Sleep stages were scored in every 20-s epoch of MEG recording by a well-trained technologist (MI) based on the following criteria. Stage W (awake state) was determined when posterior dominant alpha rhythm accounted for 50% or more of the recording with blinking or muscle artifacts. Stage N1–N2 (light sleep) was determined when posterior dominant alpha rhythm accounted for less than 50% of the recording without blinking or muscle artifacts, or when the recording included vertex sharp transients, spindles, or K-complexes. Stage N3 (slow wave sleep) was not recorded in this study because no patients or subjects showed high-amplitude slow waves in the recordings. Stage REM (rapid eye movement sleep) was not recorded in this study because no patients or subjects showed REM artifacts in the recordings.

SEFs were separately averaged for awake state and light sleep from 30 hemispheres of 15 patients with TLE and 56 hemispheres of 28 healthy volunteers. Consequently, SEF data in awake state were compared between 28 hemispheres of the TLE group (15 of 15 patients) and 47 hemispheres of the control group (25 of 28 subjects). SEF data in light sleep were compared between 19 hemispheres of the TLE group (11 of 15 patients) and 11 hemispheres of the control group (7 of 28 subjects). The data from 20 ms before to 100 ms after the stimulus onset were averaged for 100–300 times. SEF data were included for evaluation if more than 100 averages were available for each awake-sleep stage for each hemisphere. Latency corrected for subject height [ms/m] and ECD strength^41^ was evaluated for the first peak (M1, N20m) and second peak (M2)^18^.

### Statistical analysis

All statistical analysis was performed using GraphPad Prism 5 software. Two group comparison of SEFs was performed with the two-sided Mann–Whitney U test. Statistical significance was assumed for *P* < 0.05. The correlation between M1 ECD strength in the awake state and clinical variables including age, sex, side of TLE, presence of lesion, presence of HS, relationship to epileptic focus, and duration of epilepsy were also evaluated. The two-sided Mann–Whitney U test was used for categorical variables. Spearman’s ρ correlation coefficient test (2-tailed, α = 0.05) was used to analyze the correlation between M1 ECD strength and continuous variables. Outlier values defined as outside the mean ± 2 standard deviations were excluded from further analysis.

## Data Availability

Dr. Ishida had full access to all of the data in the study and takes responsibility for the integrity of the data and the accuracy of the data analysis.
